# Reviewing enjoyment in foreign language education from the perspective of positive psychology

**DOI:** 10.3389/fpsyg.2026.1784066

**Published:** 2026-03-16

**Authors:** Qunfeng Wang

**Affiliations:** School of Humanities and Foreign Languages, Xi'an University of Technology, Xi'an, China

**Keywords:** foreign language education, foreign language learning enjoyment, foreign language teaching enjoyment, positive emotion, positive psychology

## Abstract

With the emergence of positive psychology as a defined specialization, interest in positive emotions has been part of a current trend in the literature of foreign language education (FLEd). Enjoyment as a favorable feeling experienced by learners and teachers has received considerable attention of foreign language educators in a dozen years past. Scholars have widely addressed the issue of enjoyment from learner's perspective with focus on its concept, measurement, influential variables, dynamic nature, multiple roles as well as learner's enjoyment in an online foreign language learning context. Compared to the prior studies on foreign language learner's enjoyment, more studies need to be conducted to explore topics concerning enjoyment from foreign language teacher's perspective though some pioneering attempts have been made. Based on an overview of the extant enjoyment research in the field of FLEd, the present narrative review suggests to construct a hierarchical model to re-conceptualize different types of enjoyment in FLEd. Moreover, it is advisable that diversified research methods should be employed with further theoretical integration and the enrichment of research topics to unveil the complicated positive emotion of enjoyment in FLEd more extensively.

## 1 Introduction

With the advent and rapid development of positive psychology, research attention in general psychology has been shifted from negative emotion to positive emotion in the past two decades of years (Dewaele et al., 2019b). Such turn prompts the researchers and practitioners worldwide in the domain of FLEd to switch their focus from investigating negative emotions (e.g., anxiety and boredom) to positive psychological feelings arising in the process of language learning and teaching. In line with the proliferation of research on positive emotions within general psychology, the notion of positive psychology is initially brought to the realm of FLEd by MacIntyre and Gregersen (2012). It is believed that positive psychological experiences can help reduce negative arousal that results in a constriction of focus. More importantly, such experiences can produce long-term effects beyond language classroom by enhancing resilience and fortitude in the challenging periods, during which learners are able to confidently take measured risks and engage in the intriguing activities to better their language learning.

Among a range of these positive emotions in the FLEd context, enjoyment has aroused great interest of foreign language scholars. The empirical studies are initiated by the conceptualization of enjoyment in the foreign language learning environment and the investigation into the bonds between anxiety and enjoyment in the classroom from the perspective of language learners (Dewaele and MacIntyre, 2014). And then a number of research has been undertaken to explore the relevant issues, for example, the design and validation of learner's enjoyment scales (Botes et al., 2021), the contributing factors (Jin and Zhang, 2018), dynamicity (Dewaele and Meftah, 2024) and multiple roles of learner's enjoyment (Yang et al., 2024; Luo, 2025), as well as learner's enjoyment in an online situation (Bashori et al., 2021). The rich findings doubtlessly contribute to explicating learners' complicated positive psychological states when they receive FLEd and pave the way for further research on learner-centered enjoyment. Meanwhile, it is noted that the enjoyment level of foreign language teacher can not only influence teachers' instruction effectiveness but also impact learners' enjoyment and their language outcomes (Mierzwa, 2019a; Noughabi et al., 2024), so in a few years past, foreign language teacher's enjoyment has captured researchers' attention. Efforts have been made by some academics in the limited pioneering research to tackle the issues related to enjoyment that teachers undergo in the process of teaching a foreign language, e.g., development of foreign language teaching scales (Mierzwa, 2019b), influential variables of teaching enjoyment (Wei et al., 2023) and its interactions with some negative emotions (Aghayani et al., 2024). By examining these aspects, academics seek to gain a deeper understanding of how enjoyment can enhance the overall learning and teaching experience in FLEd.

Compared to other types of literature review, for example, systematic review, a scientific approach to reviewing the literature that is highly structured and protocol driven, a narrative literature review can be defined as a method to provide a comprehensive backdrop for a specific topic by synthesizing a wide array of literature into a coherent interpretation, and highlighting the core issues, trends, complexities, and controversies that lie at its heart (Efron and Ruth, 2019). In order to present the current research status on enjoyment in FLEd through the lens of positive psychology, this paper adopts a narrative literature review approach. The literature search is conducted across major databases (e.g., Web of Science, Scopus, and Google Scholar) by using such key terms as “*foreign language enjoyment*” (FLE), “*foreign language teaching enjoyment*” (FLTE) and “*enjoyment in foreign language education*.” Regarding the selection criteria, this review solely considers published empirical research articles written in English that are pertinent to both foreign language learner and teacher enjoyment. Consequently, conference papers, review articles, and non-empirical works are excluded. The objective of this narrative review is to synthesize and analyze the existing studies concerning enjoyment experienced by foreign language learner and teacher in a coherent and comprehensive manner, summarize the progression and major findings over the past 12 years and provide guidance for future enjoyment research within the context of FLEd.

## 2 Enjoyment in positive psychology

The concept of enjoyment has been explained in detail by many scholars with the development of positive and educational psychology theories. Seligman (2012) proposes PERMA model (Positive Emotion, Engagement, Relationships, Meaning, and Accomplishment) from the perspective of positive psychology. Three aspects for positive psychology research are put forward: valued subjective experience, positive individual traits, and the civic virtues and the institutions. As a positive subjective experience, enjoyment refers to the good feelings that can result in individual growth and long-term happiness when people break through the limits of homeostasis, which is differentiated from pleasure that can satisfy the physical needs for hunger, bodily comfort etc. (Seligman and Csikszentmihalyi, 2000). Similarly, enjoyment is described as a kind of positive emotion that differs from the pleasure experience in that it could help enhance progression or challenge limits (Boudreau et al., 2018). Moreover, enjoyment is characterized by some additional dimensions that include an intellectual focus, heightened attention, and optimal challenge whereas pleasure can be experienced simply by performing an activity or completing an action (Boudreau et al., 2018). This interpretation of enjoyment is also echoed by Dewaele and MacIntyre (2016), defining it as a complex emotion involving challenge and perceived ability that enable the individuals to drive for success by the completion of challengeable tasks. In other words, enjoyment can be experienced in the situations where people exceed the needs already met to acquire the accomplishment beyond their expectations. But pleasure, a much simpler feeling, can occur when something likable is happening. In the field of educational psychology, the control-value theory is constructed to provide an integrative framework for analyzing the antecedents and effects of emotions experienced in achievement and academic settings (Pekrun, 2006; Shao et al., 2019). According to it, achievement emotions lie in three dimensions: object focus (the activity itself vs. the outcome), valence (positive vs. negative), and activation (deactivation vs. activation), in which enjoyment is defined as an activity-related, positive and activating achievement emotion experienced through the learning activities. Taken as a whole, a common practice to portray enjoyment as a positive emotion in both positive and educational psychology in the previous studies is to make a comparison and contrast between similar or opposite concepts of human emotions. It can be concluded that in the light of positive psychology, enjoyment is a favorable feeling or positive emotional experience that can facilitate personal development. Such nature of enjoyment makes it possible to introduce and adapt positive psychology theories to emotional studies on FLEd.

## 3 Foreign language enjoyment

### 3.1 Concept of foreign language enjoyment

The conceptualization of FLE follows after MacIntyre and Gregersen (2012) provide some ideas about emotion in foreign language learning from the perspective of positive psychology. Dewaele and MacIntyre (2014) firstly use the term of FLE when enjoyment and anxiety in a foreign language learning context are discussed. FLE, a complex emotion associated with the interaction between challenge and perceptual ability, is further described as a separate dimension from foreign language classroom anxiety (FLCA), although FLE is possibly accompanied by FLCA (Dewaele and MacIntyre, 2016). Besides, two dimensions of FLE are revealed. The social dimension emphasizes the satisfaction that language learners gain in a positive foreign language classroom, for example, friendly learning environment, mutual support from learners, teachers' emotional support like encouragement, and exciting classroom activities. The second dimension is related to private thoughts and feelings, for instance, the pride and satisfaction arising from confronting challenges and removing difficulties (Dewaele and MacIntyre, 2016; Dewaele and Li, 2022). A more formal definition of FLE is given by Dewaele (2022), stating that FLE is the emotion that can fuel the foreign language learning process and help improve foreign language performance. According to it, enjoyment in a foreign language learning environment differs from pleasure in that challenge, hard work as well as a sense of fulfillment are implied at the successful completion of arduous language learning related activities. It can be concluded that the concept of FLE at least includes three respects. First, it focuses on language learners instead of teachers or instructors. Second, it is a positive emotional experience in the process of foreign language learning, which is suggested to be examined together with the negative emotions like anxiety or others. Third, FLE highlights the various aids that enjoyment can offer for successful language learning through confronting and completing challengeable language learning tasks. With the introduction of the concept of enjoyment to FLEd, researchers are provided with new insights and more topics to explore the relationships between emotions and foreign language learning from the perspective of positive psychology. However, there is a tendency that foreign language enjoyment, foreign language learners' enjoyment, foreign language learning enjoyment are used interchangeably in the existing documents to refer to the enjoyment experienced by language learner in the process of learning a foreign language. This can cause confusion because the concept of foreign language enjoyment can be explained as a wider terminology that can be extended to include the enjoyment experienced by foreign language teachers. Therefore, there is a need to redefine the three terms academically in future research.

### 3.2 Measurement of foreign language enjoyment

The growing scholarly interest in FLE has prompted the development of various instruments tailored to different research contexts. Since Dewaele and MacIntyre (2014) design the 21-item *Foreign Language Enjoyment Scale* (FLES) by adapting seven items from Ryan et al. (1990) Interest/Enjoyment subscale, the measurement of FLE has undergone notable refinement, particularly in terms of its dimensional structure. A key development lies in the evolving dimensionality of the construct. While the initial 21-item scale is later reduced to 14-item to constitute a two-factor structure comprising FLE-Social (positive feelings from encouraging peers, supportive teachers, and a positive environment) and FLE-Private (personal sense of accomplishment; Dewaele and MacIntyre, 2016), subsequent studies have yielded divergent dimensional structures. For instance, Dewaele and Dewaele (2017) propose a three-factor 10-item version adding a peer-controlled vs. teacher-controlled dimension, whereas Li et al. (2018), adapting the scale for the Chinese context, develop an 11-item *Chinese Version of FLE Scale* (CFLES) with three distinct dimensions: FLE-Private (enjoyment from fun, accomplishments, and interesting learning experiences), FLE-Teacher (enjoyment derived from teachers' supportive attitudes and pedagogical practices), and FLE-Atmosphere (enjoyment from a friendly learning environment). These variations may reflect contextual differences—for example, the greater emphasis on teacher-related factors in the Chinese version could be attributed to the teacher-centered nature of many Chinese EFL classrooms. More recently, Botes et al. (2021) constructs a nine-item *Short Form of the Foreign Language Enjoyment Scale* (S-FLES), which is tested as a reliable and valid three-factor hierarchical model with FLE as a higher-order factor and Teacher Appreciation, Personal Enjoyment, and Social Enjoyment as lower-order factors. This progression from unidimensional to multidimensional and hierarchical conceptualizations suggests an increasingly nuanced understanding of the construct. Despite these developments, the majority of FLE studies continue to draw upon Dewaele and MacIntyre's (2014) 21-item FLES, often with adapted or translated versions suited to specific learning contexts and research objectives (Elahi Shirvan et al., 2021; Jin and Zhang, 2018; Wang and Wang, 2024; Xethakis et al., 2025).

### 3.3 Factors contributing to foreign language enjoyment

As a personal psychological experience that contributes to constituting individual's sophisticated emotional system, FLE are affected by a wide range of variables that can be mainly categorized into three groups: learner-related variables, teacher-related variables and social variables. Some learner-related variables, such as age, gender, education level, language proficiency, multilingualism, are also considered as the demographic or sociodemographic variables. It is found in the existing literature that older language learners could undergo more FLE than younger learners (Dewaele et al., 2018). However, the relationship between FLE level and age doesn't appear as a linear pattern (Dewaele and Dewaele, 2017), which is further supported by the finding that learners' FLE level is increased from first to second grade of a secondary school, but sharply decreased in the third grade (Mierzwa, 2018). The sudden reduction of FLE of the learners in the third grade can be ascribed to the enhanced pressure from examinations and the discouragement caused by the demanding learning materials. The results of some studies indicate that female learners score higher than male learners in terms of FLE level (Bensalem, 2021; Huang and Jiang, 2022), but gender difference is not revealed in some other research (Guo, 2021; Yeşilçinar and Erdemir, 2022). Moreover, a higher education level, a higher language proficiency, and mastery of several languages can lead to a higher FLE level as well (Dewaele and MacIntyre, 2014). For example, Botes et al. (2020) discover that foreign language learners with a higher level of multilingualism and self-perceived foreign language proficiency experience a higher level of enjoyment and lower level of anxiety. But this is inconsistent with the finding of Dewaele et al. (2018) that no significant relationship is revealed between the degree of multilingualism and FLE. It is further emphasized that learner related variables, such as more positive attitude toward foreign language learning and foreign language teacher, more time spent on oral practice, and a higher foreign language proficiency, are linked to higher level of FLE. Additionally, teachers' role in exerting positive influence on learners' FLE is uncovered that more frequent use of foreign language by teacher can lead to more learners' FLE, which is in line with the findings by Dewaele and Dewaele (2020) and Dewaele et al. (2023).

A great number of other teacher-related contributors (e.g., teacher personality traits including openness, extroversion, and agreeableness; teacher characteristics such as friendliness, humor, patience; teaching methods; teacher appreciation; positive feedback and encouragement) are confirmed to be positively associated with FLE by a number of studies (Jin and Zhang, 2018; Kim, 2023; Shima et al., 2020). For example, (Yeşilçinar and Erdemir 2022) report that a teacher's role in fostering FLE extends beyond simply being an expert in the language. Their research highlights the considerable impact of teacher characteristics like friendliness, support, and encouragement. These findings are in line with Phuong (2025) conclusion that teacher appreciation is a key contributor to positive emotional experiences for language learners. It is also pointed out that teaching methodology (e.g., games and interesting activities, interesting topics, rich teaching techniques, and learner-centered teaching) can be positively influential to learners' FLE. This is similar to Yang (2021) finding that teaching strategy and attractive classroom activities are indispensable sources of FLE. Compared to learner and teacher related variables, social variable is more abstract with its focus on the classroom learning environment or atmosphere. Dewaele and MacIntyre (2016) find that a good classroom learning atmosphere among teacher and peers that is created by the combination of positive feelings, encouraging peers, nice teachers, and a supportive environment is a crucial factor in producing enjoyment. This result is echoed by Li et al. (2018), Mierzwa (2019a), and Mierzwa (2019b), underscoring the connection between FLE and the classroom atmosphere generated by peer interaction and positive engagement. Some scholars examine those variables from the internal and external perspectives with the adoption of dichotomy, so the external is extended to cover factors irrelevant to classroom education. For instance, institutional variables such as facilities and location of the institution are reported to have a significant impact on FLE (Yeşilçinar and Erdemir, 2022).

To sum up, the majority of the variables explored so far play a positive role in strengthening learners' FLE, but the impact of a few variables (e.g., gender and multilingualism) on FLE is not consistent. This possibly results from the sample size or other potential factors worthy of being investigated, for example, culture. After all, the emotion experienced in foreign language learning is contextually situated and could be impacted by the sociocultural environment. Besides, no matter which factorial structure of FLE is accepted, three or two component based pattern, it cannot be denied that those variables are not independent and rather, collaboratively influence learners' FLE despite how they are labeled.

### 3.4 Dynamic nature of foreign language enjoyment

Complex dynamic systems theory, which views language as a dynamic system of interacting subsystems, has been introduced to investigate the dynamic nature of FLE. The dynamicity of FLE is examined basically from the longitudinal perspective that is focused on FLE changes on a group of learners over a periodic time (Elahi Shirvan et al., 2021) and from the idiodynamic perspective that is concentrated on FLE momentary fluctuations of individuals (Boudreau et al., 2018). Utilizing the pseudo-longitudinal method to evaluate the dynamics of FLCA and FLE of 189 foreign language students aged from 12 to 18, Dewaele and Dewaele (2017) find that both FLE and FLCA evolve over time and various psychological and socio-biographical variables lead to the fluctuation in FLCA and FLE. Similarly, by using a latent growth curve modeling to investigate FLE and FLCA changes of 367 Iran undergraduate students, another longitudinal study by (Elahi Shirvan and Taherian 2018) indicates that FLE and FLCA can be regarded as a dynamic system and develop over time. It is reported that the interactive influence of various elements, such as teachers' feedback, peers' support, material quality, class performance, makes the development of learners' FLE and FLCA unpredictable. Moreover, FLE and FLCA self-organize into attractor states in which learners feel a stable state of anxiety and enjoyment. And they are feedback sensitive and may alter dramatically. The FLE and FLA alteration during a period is also supported by the findings from the longitudinal investigation by Pan and Zhang (2023). It is revealed that FLE is not as stable over time as FLA because a quantity of motivational factors (e.g., ought to L2 self, ideal L2 self, motivated behavior) can contribute to the variance of FLE over time. The idiodynamic method, initially introduced by MacIntyre and Legatto (2011) to investigate the moment-to-moment changes of SLA variables, is also used to examine the dynamics of FLE. In the study by (Elahi Shirvan and Talebzadeh 2017), seven female university students are required to self-rate their moments of enjoyment experienced in the conversation on both simple and difficult topics. The results reveal that the conversational topics can result in the dynamicity of enjoyment not only within individuals, but also across individuals intra-personally. Talebzadeh et al. (2019) explore the mechanisms and dynamics of enjoyment contagion in a FLEd context with the employment of the idiodynamic method in five dyadic teacher-student interactions. It is concluded that the major mechanism of enjoyment contagion in these interactions is the automatic mimicry of mutual verbal and non-verbal behaviors between learners and the teacher, which renders the process of enjoyment contagion dynamic.

More recently, there are also other methods being developed in an attempt to unravel the complex dynamic system of individual FLE. The ecological momentary assessment, together with the idiodynamic approach, the enjoy-meter, weekly journals and open-ended interviews, is used to explore the dynamism of different facets of FLE experienced by two learners across seconds, minutes, weeks, and months in a ESL learning context (Elahi Shirvan et al., 2020). The findings indicate that the enjoyment moments are unique to each learner and the change of the main ecological drivers including private and social factors can be tremendously influential. Each ecological timescale can help formulate different enjoyment pattern for each learner, but learners could experience similar pattern of enjoyment under the influence of the same ecological factors. Based on the complex dynamic systems theory, Li (2024) reports a non-linear dynamic correlation pattern between FLE and FLA over a short window of time with the use of the emotion dynamic modeling and such pattern is claimed to be individualized. In line with the findings of the previous research (De Ruiter et al., 2019; Elahi Shirvan and Talebzadeh, 2020), this study also finds that both internal and external variables can cause fluctuating changes of FLE and FLA.

Taken as a whole, scholars have shown great interest on the investigation into the dynamic mechanisms of FLE and how FLE changes within individuals and groups of learners to gain a better understanding of the interactions between varieties of emotions in the light of positive psychology. With the frequent employment of longitudinal and the idiodynamic methods, the underlying factors that could potentially cause the fluctuations of FLE as a dynamic system are disclosed. Enlightened by the adaptation of complex dynamic theory into FLEd, researchers tend to develop and apply diversified methods and theories to present more convincing evidence on FLE dynamicity to help learners improve learning efficiency and instructors enhance their teaching in the foreign language context.

### 3.5 Multifaceted roles of foreign language enjoyment

The capacities to experience positive emotion, for example, joy, defined as a psychological response to a good object, usually a positive event or circumstance (Watkins et al., 2017), can be construed as fundamental human strengths that produce multiple and interrelated benefits. One of its assumed functions is that the lingering effect of negative emotions can be corrected or undone by positive emotions (Fredrickson, 2001). Inspired by such hypothesis, scholars in the field of FLEd have endeavored to explore the relationship between enjoyment as a typical positive emotion and anxiety as a regular negative emotion. The pioneering attempt is made by Dewaele and MacIntyre (2014), who report a significant negative correlation between FLE and FLCA, but claim that they are independent emotions because the lack of one does not automatically imply a high level of the other. This finding is confirmed by a number of studies, suggesting that anxiety and enjoyment are separate dimensions whereas building more FLE experiences can lead to the decrease of FLCA (Bensalem, 2021; Chen et al., 2023; Dewaele and Alfawzan, 2018; Khan et al., 2024; Li, 2019). However, in the study conducted by Boudreau et al. (2018), a highly dynamic relationship between enjoyment and anxiety is found as the correlation varies from negative to positive and then to zero. That is to say, the two emotions can move in converging patterns or at other time show divergent trajectories, even work independently of one another to follow unpredictable trajectories. The finding is partially supported by Dewaele et al. (2019a), who find a weak positive correlation between participants' FLE and FLCA. It is possible that learners with higher FLE can also experience slightly more FLCA due to a heightened emotional state. The inconsistent findings on the relationship between FLE and FLCA indicate the complex of learners' emotions in the process of foreign language learning. A succession of emotions, positive or negative, may impact each other and be influential to foreign language learning. Therefore, when the role that a certain emotion like enjoyment plays on language learning is evaluated, other emotions are strongly suggested to be considered simultaneously to better interpret learners' psychological states and their learning behaviors.

Another role of enjoyment on foreign language learning that has been uncovered lies in its impact on learner's achievement. The majority of the present studies have found a positive relationship between FLE and foreign language achievement (Botes et al., 2020; Jin and Zhang, 2018; Piechurska-Kuciel, 2017). In the study that examines the effect of FLE and FLCA on foreign language performance among learners from two London secondary schools and Saudi EFL learners, Dewaele and Alfawzan (2018) report that higher levels of FLE are linked to significantly higher English achievement and higher levels of FLCA are linked to lower performance. In other words, learners who experience more enjoyment and less anxiety are more likely to perform better in language learning. A similar conclusion is made by Jin and Zhang (2018), according to which, enjoyment of foreign language learning has a direct and positive effect on learners' test scores. When learners enjoy learning a foreign language, the intrinsic motivation can be triggered to improve their language performances. In a follow-up research, Li (2019) puts forward the concepts of perceived achievement and actual achievement in foreign language leaning and finds that FLE is positively related to both perceived achievement and actual achievement of learners. However, an inconsistent finding reported by Li and Han (2022) reveals that FLE and foreign language boredom (FLB) can predict perceived achievement rather than actual achievement. This conflicting conclusion can be understood as such that FLE may not be the only factor that influence learners' language achievement. Therefore, there is the tendency to evaluate the relationship between FLE and language achievement with consideration of other psychological variables, such as learner engagement (Guo, 2021; Tsang and Dewaele, 2023), FLB (Tsang and Davis, 2024; Wang and Li, 2022), grit (Fathi and Hejazi, 2024; Jin, 2024; Zou et al., 2025), willingness to communicate (Demir and Okyar, 2021; Fathi et al., 2023; Lin and Wang, 2025). Those emotional factors are more or less related to each other and constitute a complicated psychological network to impact foreign language learning positively or negatively. It is expected that in future studies more individual differences can be taken into account to present persuasive evidence to lift the curtain on the complex of positive psychology.

Due to the complex and dynamicity of emotion, due attention has also been paid to the mediating role of FLE among different variables. FLE can function as a bridge or a mediator that connects the learning process and the learning outcomes, which means that enjoyment experienced in learning a foreign language can influence learners' motivation, engagement, or other potential contributors and ultimately strengthen their language achievements. In the study investigating the mediating effect of FLE, Wei et al. (2019) discover that FLE can mediate the relationship between grit and performance, that is, grit can generate a positive effect on foreign language performance by increasing learners' FLA level. Such mediating role is also reported by Wang et al. (2023), claiming that FLE can exert a significant and positive influence on learners' engagement in class, which, in turn, positively impacts their learning outcomes. Likewise, the indirect effect of FLE is detected in a more recent study, suggesting that teacher support and grit could affect leaners' willingness to communicate through the mediation of FLE (Yang et al., 2024). It means that gritty learners or learners who receive much support from teachers can possibly experience more enjoyment in the process of foreign language learning, thus making them engage in learning interactions more enthusiastically. With the adoption of quantitative analysis, Liu et al. (2024) concludes that there is a strong positive correlation between the ideal L2 self and learners' informal digital learning of English, which is partially mediated by FLE. To conclude, current research has shed lights on the role of FLE on mediating the relationship among varieties of individual differences in foreign language learning environments. The revelation of FLE as a mediator provides researchers with novel insights into the complex system of foreign language learners' emotions.

### 3.6 Foreign language enjoyment in an online learning context

The advancement of technology makes online learning an indispensable part of FLEd, which encourages scholars to be dedicated to investigating FLE in an online learning context. Following the research focus of enjoyment in in-person classes or offline classes, scholars have discussed the similar topics, such as the development of *Online Foreign Language Enjoyment Scale* with Teacher, Private, -Interaction, and Competence dimensions (Wang et al., 2021); the relationship between online FLE and FLA (Atigh et al., 2023; Resnik and Dewaele, 2023); the influential factors of FLE, for example, the demographic variables (Wang and Jiang, 2022), the learner-internal and teacher-related variables (Yuan, 2023), personality traits and other individual differences, e.g., autonomy, trait emotional intelligence (Resnik and Dewaele, 2023), grit, online learning self-efficacy and online learning engagement (Derakhshan and Fathi, 2023), anxiety, enjoyment, boredom and engagement (Wang and Hui, 2024). Fattahi et al. (2023) found that boredom is a considerably stronger predictor of willingness to communicate (WTC) than FLE although FLE can mediate the relationship between boredom and WTC. This finding marks a departure from earlier research conducted in in-person classroom settings. One of the downsides of online FLEd is then pointed out that the limited impact of FLE may result from the lack of face-to-face interaction and socialization. But solutions concerning the removal of such disadvantage is not proposed, so future research is expected to explore specific strategies to reduce online boredom and increase online FLE to facilitate online language education. Besides, the issue of whether online FLE is correlated with learners' achievements is addressed. Wang and Li (2022) find that both learners' actual performance and self-perceived performance are significantly positively correlated with FLE and negatively correlated with FLA and FLB in an online-based learning context. That is to say, the more positive emotions learners experience in online classes, the less anxiety and boredom they may undergo, the better achievement they can make. In addition, dynamicity of online enjoyment and emotion regulation are investigated. With the employment of the idiodynamic approach, changes of group-level enjoyment over 18 weeks by completing collaboratively a series of online writing tasks are observed (Zhang et al., 2024). It is reported that online group-level enjoyment fluctuates within each of three writing sessions and possibly stay longer in the later session when group members' collaboration advances. Among the three types of emotion regulation identified, socially shared regulation is more depended on by learners to achieve and sustain the dynamic evolution of online group-level enjoyment compared to self-regulation and co-regulation. Finally, scholars tend to deploy questionnaire or quasi-experimental approach to compare and contrast FLE in in-person and online classes. FLE in online class can be sharply reduced when compared to that in in-person class because of the reduced group laughter, physical isolation, less interactive teaching etc., but it wouldn't disappear completely due to the increased flexibility and autonomy of online classes (Resnik et al., 2023). However, Bai (2024) reports that the experimental group who uses an online language learning platform (Rosetta Stone) with in-class instructions shows significant improvement on their listening, speaking, FLE, but demonstrates less FLA than the control group who receives only the regular in-class instruction. Generally speaking, online FLE shares some similarities with FLE in-person class concerning the influential factors, dynamic nature, the role in the process of language learning etc. It should be noted that the lack of face to face interactions among students and teachers, the unfamiliarity with the online learning tools as well as other potential factors can lead to the lower level of FLE in an online learning environment, so future research is suggested to focus on the reduction of such downsides to help increase learners' online FLE to facilitate their language learning.

## 4 Foreign language teaching enjoyment

### 4.1 Concept of foreign language teaching enjoyment

As what has been well documented, teachers' psychological states have impact on FLE that students undergo in a foreign language classroom. Such finding arouses researchers' interest on the investigation into enjoyment acquired by foreign language teachers. Mierzwa (2019b) firstly uses the term of FLTE to access teacher's enjoyment level in the process of teaching a foreign language. In the successive research, FLTE is further classified to include teachers' personal enjoyment in a foreign language teaching context, social enjoyment in a foreign language teaching classroom and enjoyment gained by being appreciated by students (Proietti Ergün and Dewaele, 2021). Though, in the past few years, FLTE has been one of the concerns in FLEd, a clear-cut and formal definition of FLTE is not given. In the existing literature, two terms, namely foreign language teacher enjoyment and foreign language teaching enjoyment, are being used interchangeably. But the former can be understood as a broader concept that includes both teaching enjoyment and non-teaching enjoyment, for example, teachers' enjoyment arising from learning a foreign language. It was not until 2023 that FLTE was defined academically. It refers to teacher's positive joyous emotional experience in teaching a L2 language, which may result from teacher-external and/or teacher-internal variables (Solhi and Shirvan, 2023). Noughabi et al. (2024) further define it as the positive emotion experienced by teachers in a foreign language teaching context where challenges that possibly impact foreign language teachers' psychological growth need to be coped with. FLTE appears to be conceptualized in the same way as FLE is defined, based on which three teaching-related components are underlined: positive emotion, teaching environment, and challenges confronted by teachers. Such an evident definition of enjoyment from teachers' perspective is needed to eliminate academic confusion and assist researchers to investigate the recently focused positive psychological state related to foreign language teacher more effectively.

### 4.2 Measurement of foreign language teaching enjoyment

As the counterpart of FLE, FLTE is evaluated by the adaptation of the FLE scales to the foreign language teaching environment. On the basis of FLES by Dewaele and MacIntyre (2014), the first *Foreign Language Teaching Enjoyment Scale* is designed by Mierzwa (2019b), which includes 17 items constructed in a positive way, for instance, “I enjoy teaching a foreign language.” (Proietti Ergün and Dewaele 2021) present a nine-item scale adapted from S-FLES developed by Botes et al. (2021) to measure teachers' personal and social enjoyment in the classroom as well as enjoyment from students' appreciation. Adapting (Proietti Ergün and Dewaele 2021) and Dewaele and MacIntyre's (2014) survey items to suit Japanese context, Onodera (2023) develops a new 15-item scale that is composed of three dimensions: personal enjoyment of teaching (e.g., I enjoy teaching English), student appreciation of classroom teaching (e.g., My students appreciate the effort that I put in teaching English), and social enjoyment of teaching (e.g., My students and I help and respect each other in class). Apart from the aforementioned scale, a different tool, *Q-Sort Technique*, is employed by Thumvichit (2022) to identify viewpoints of EFL teachers at university level on enjoyment in their professional context. The Q sort of 44 statements related to enjoyment of foreign language teachers indicates three factors contributing to teacher's enjoyment: classroom engagement, career value and social interaction. To date, in the limited literature exploring FLTE, the scale by Proietti Ergün and Dewaele seems to be popular and even used directly without modification on the ground of its high internal consistency (Aghayani et al., 2024; Habib et al., 2025; Solhi and Shirvan, 2023; Yang et al., 2023). It is expected that diversified tools can be utilized to collect data on FLTE in future research. At least, while using the previous foreign language teaching scales, researchers are suggested to adapt them to be suitable to the participants' learning contexts, because enjoyment experienced by foreign language teacher is also socio-culturally situated.

### 4.3 Factors contributing to foreign language teaching enjoyment

Similar to FLE, FLTE is also assumed to be dependent on other variables to contribute to foreign language teachers' professional development and psychological growth. These influential variables are being investigated by scholars mainly through five lenses in the current studies. From the perspective of demography, the variables, such as gender, age, education background, school type, teaching experiences, teaching experiences (years), place of residence, and type of foreign language being taught, have no significant or limited effect on FLTE (Mierzwa, 2019b; Onodera, 2023; Proietti Ergün and Dewaele, 2021). The influential factors on FLTE are also examined by categorizing the enjoyment into three dimensions: personal enjoyment, social enjoyment and student appreciation, but only a few studies available have discussed how each dimension is associated with teacher's overall FLTE. Solhi and Shirvan (2023) find that Turkish EFL university teachers show the highest level of social enjoyment and the lowest of personal enjoyment. To be specific, social variables, e.g., being with colleagues as an external factor, have the most supporting role in foreign language teachers' enjoyment compared to teachers' personal or internal factors, e.g., attitudes toward profession. Based on the data collected from the open ended questionnaire, Noughabi et al. (2024) report 10 factors enhancing FLTE, for example, motivated students, positive workplace environment, collaboration with colleagues, sufficient technological facilities etc. Though these variables are not classified into different groups with the construction of a hierarchical pattern, many of them can be considered as teacher-external social factors. In line with the pervious finding, it is highlighted that social and institutional support among colleagues, parents, authorities, and school administrations may play an essential role in enhancing FLTE. However, this conclusion is contradictory to the finding of Onodera (2023), arguing that student appreciation and personal enjoyment might be more effective to empower foreign language teacher rather than social enjoyment. The inconsistency might be owing to the cultural variance of the participants involved, different sample sizes, or other factors that have not been documented. Therefore, more evidence need to be presented in future research to give a further explanation on the conflicting results.

Besides, researchers also link FLTE to personality traits or personality related variables and some other individual differences. The complicated interactive relationships among them and their potential influence on teachers' FLTE have been depicted. Resilience and wellbeing is found to be the significant predictors of FLTE and resilience can be directly associated with wellbeing to function as a protective system for wellbeing (Derakhshan et al., 2022; Proietti Ergün and Dewaele, 2021). The impact of wellbeing on FLTE is confirmed by Azari Noughabi et al. (2022), suggesting that teachers who are more capable of regulating their emotions are more likely to experience profession happiness, which in turn leads them to gain more enjoyment. This finding is supported by Alkarkhi and Hmouma (2025), reporting that among four variables: wellbeing, resilience, work environment, and job satisfaction that collectively enhance FLTE, only wellbeing significantly predicts FLTE. Other personality traits identified to be closely connected to FLTE are teacher's grit and engagement. The findings reveal a significant positive relationship between grit and FLTE among foreign language or L2 teachers, which means that gritty teachers may undergo more enjoyment in the process of teaching and conversely, the more enjoyment they experience, the grittier they could become (Zhang et al., 2023). This positive correlation is echoed in the study conducted by Derakhshan et al. (2022). Moreover, the study unveils a positive relationship among grit, resilience and wellbeing. With regard to the role of engagement, a bidirectional relationship between work engagement and FLTE is found (Zhang et al., 2023), which can be interpreted as such that higher FLTE predicts increased work engagement and vice versa. Additionally, the cross-lagged relations discovered indicate that teachers with higher levels of self-efficacy experience more FLTE, grit, and engagement. Being humorous doesn't always lead to an increase of FLTE level, which has been pointed out in Solhi and Shirvan (2023) study. It is disclosed that L2 teacher's self-enhancing, affiliative humor styles and self-defeating humor style are positively correlated with their classroom enjoyment. The use of benign humor can be helpful for L2 teacher to establish good social bonds with colleagues and students, thus resulting in higher levels of social enjoyment and student appreciations. In contrast, the aggressive humor style can reduce both student enjoyment and teacher enjoyment. Similarly, grounded on categorizing motivation into autonomous motivation and amotivation, Onodera (2023) finds that FLTE shows a significant positive correlation with autonomous motivation driven by internal factors (personal fulfillment and growth and student-centered altruistic desire) and negative correlation with amotivation (inadequate stakeholder understanding and heavy workload).

Since positive emotions have been proven to not only be conducive to build personal resources but also help undo the arousal of negative psychological emotions (Fredrickson, 2001), foreign language teachers' negative feelings are taken into account when FLTE is investigated. Aghayani et al. (2024) report a significant negative relationship between FLTE and burnout and a significant positive relationship between FLTE and resilience. Therefore, the supportive educational contexts and improvement of teachers' social and emotional competence are suggested to enable teachers to enhance their FLTE and reduce burnout by creating favorable classroom teaching atmosphere and foster resilience. Another burnout-related study that focuses on the mediation role of FLTE is undertaken by Yang et al. (2023), who find that mindfulness is negatively related to EFL teachers' burnout and positively linked to their work engagement. Furthermore, such relationships can be partially mediated by FLTE. A positive feedback loop is therefore confirmed, in which more enjoyment in the profession found by mindful EFL teachers can possibly lead to increased work engagement and reduced burnout, which in turn can enhance teachers' enjoyment and overall job satisfaction.

In sum, FLTE level can be ascribed to a wealth of variables in the existing literature, for example, demographic variables, internal and external variables, personally-related variables, other individual differences (non-personally related), and negative emotions. Scholars have made initial attempts to reveal the complicated relationship among these factors that construct teacher's psychological network from the perspective of positive psychology. However, compared to FLE studies centered on foreign language learners, research on the influential factors of teachers' FLTE is not sufficient and the evidence provided is not rich. More potential psychological or non-psychological variables contributing to the enjoyment experienced by foreign language teachers are needed to be investigated in future research.

## 5 Suggestions for future research

### 5.1 Conceptual development of enjoyment in FLEd

**Figure 1:**
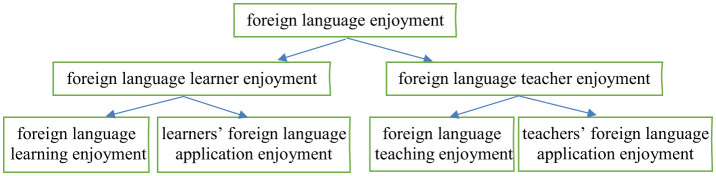
Diagram of enjoyment in FLEd (Source: original).

Previous studies have predominantly conceptualized FLE within classroom-based learning and teaching contexts (Dewaele and MacIntyre, 2016; Dewaele and Dewaele, 2017), but the role of authentic language use outside pedagogical settings as a source of enjoyment remains underexplored. It can be assumed that when learners successfully apply a foreign language in real-world contexts, such as serving as volunteers providing foreign language services for international conferences or cultural exchanges, enjoyment can be experienced, which in turn may facilitate their language learning to a large extent. Likewise, it can be hypothesized that when teachers engage in language application activities beyond teaching—for example, translating a book—they may also undergo enjoyment, potentially enhancing their wellbeing and professional practice. However, this form of enjoyment, arising from the actual use of a foreign language rather than its instruction or acquisition, has not been addressed in the existing literature. To fill this gap, the concept of *foreign language application enjoyment* is suggested to be introduced, defined as a positive psychological emotion experienced by learners and teachers in the authentic use of a foreign language. This concept extends prior frameworks by acknowledging that enjoyment in FLEd is not confined to pedagogical settings but can also emerge from real-world language use. The concept can be further differentiated from the perspective of learners and teachers: *learners' foreign language application enjoyment* and *teachers' foreign language application enjoyment*. To integrate this new dimension with the existing conceptualizations and avoid terminological confusion, a hierarchical model of FLE is proposed (see Figure 1). In this model, *foreign language enjoyment*, a more general academic terminology that refers to the enjoyment experienced by both foreign language learners and teachers in general, serves as an overarching construct encompassing both *foreign language learner enjoyment* and *foreign language teacher enjoyment*. The former comprises *foreign language learning enjoyment* gained in classroom learning or self-study and *learners' foreign language application enjoyment* experienced when learners use the language to complete real-world tasks. Similarly, *foreign language teacher enjoyment* consists of *foreign language teaching enjoyment* (positive feelings arising from the teaching process) and *teachers' foreign language application enjoyment* (positive emotions experienced when foreign language teachers, often non-native speakers, actively use the target language in application-oriented activities). This hierarchical framework addresses the need for greater conceptual clarity by systematically categorizing distinct yet interrelated sources of enjoyment. Researchers may access enjoyment from different angles, so it does not necessarily mean that a rigid conceptual boundary with regard to enjoyment in FLEd should be set and a consensus may not be reached. However, a systematic categorization of enjoyment and clarification of different concepts of enjoyment allow researchers to target specific dimensions of FLE, such as learning enjoyment, teaching enjoyment, or application enjoyment and help avoid academic ambiguity when positive psychology is adapted to the domain of FLEd.

### 5.2 Further theoretical integration and adoption of diversified research methods

Owing to the complex, dynamicity and sociocultural nature of enjoyment in FLEd, more theories can be deployed and integrated to guide the empirical studies. Though the factors influencing foreign language learners' and teachers' enjoyment level are classified from different lights in the research, it should be noted that there is likelihood that one variable can fall in two groups simultaneously with the focus shifted. For example, concerning foreign language learning enjoyment, friendliness as one of the inclusive teacher characteristics can be a subset of teacher-related variables that help increase learners' enjoyment whereas it can also be referred to as a social element because such character of a teacher could be conducive to the creation of a favorable classroom atmosphere. Likewise, relationships with colleagues, within the study of foreign language teaching enjoyment, can be conceptualized as either a social variable or a personal variable. Moreover, as is revealed, varieties of personality traits, non-personally related individual differences and other possible variables are connected with each other to impact the enjoyment of both learners and teachers. These can be the evidence to support that human emotion is a complicated system, so when enjoyment in FLEd is investigated, it is necessary to take a holistic view to assess such complex of emotional system to better demonstrate learner's and teacher's psychological states (Griffiths and Soruç, 2020). In addition, the enjoyment experienced by foreign language learner and teacher is contextually suited and presumably impacted by their sociocultural environments. For instance, an Asian EFL learner can gain more enjoyment by the adoption of memorization strategy in the language learning process because this traditional strategy is highly praised in the Confucian culture. However, the frequent use of such strategy by a Western learner who learns Chinese as a foreign language might lead to lower level of enjoyment in that memorization is taken as being mechanical in the Western settings. This assumption that sociocultural environments can influence enjoyment is suggested to be verified with the introduction of the theory of social-ecological context. Similar to the fluctuations of foreign language learning enjoyment, it is hypothesized that FLTE can alter on a moment-to moment or a periodic base, but as a newly explored research issue, evidence on teacher's enjoyment change is scarce. Therefore, it is strongly suggested that the complex dynamic systems theory should be integrated with the positive psychology theories to uncover the dynamicity of teacher enjoyment with the employment of the longitudinal method and the idiodynamic method.

When it comes to methodology, diversified research methods are suggested to be deployed. As what has been disclosed in the current literature, the quantitative methods are essentially used to collect data of enjoyment experienced by a group of language learners or teachers while the qualitative methods are concerned with the psychological states of individuals and their subjective viewpoints. It is widely recognized that the strengths of the quantitative and qualitative method can be combined and the best of both paradigms can be presented (Dörnyei, 2007), so more mixed methods studies can be expected so that the qualitative and quantitative data can be triangulated each other to enhance the research validity (Wang, 2023). Besides, the development or adaptation of Likert-scale questionnaires for measuring enjoyment in quantitative research should take into account the sociocultural contexts of participants, given that the sources of enjoyment can vary substantially from one context to another. The data collected by the direct use of the existing scale without suiting the participants' learning or teaching environments may not be persuasive enough to comprehensively account for the enjoyment-related issues in FLEd.

### 5.3 Research topic expansion

With the reconceptualization of different types of enjoyment in FLEd and further theoretical integration, the research topics can be expanded to a large extent. First, since the existing FLE research has predominantly focused on classroom-based learning and teaching contexts, learners' and teachers' foreign language application enjoyment, the newly-proposed concepts by the author, are worthy of being investigated. Future research is suggested to focus on the topics related to the measurement, the contributing factors (demographic variables, personality and non-personality variables) of such enjoyment, their relations with foreign language learning and teaching enjoyment and how they are combined to contribute to foreign language learner and teacher enjoyment.

Second, the previous studies have identified various individual difference variables influencing foreign language learning and teaching enjoyment, but the scope of these investigations remains largely limited to personality traits, leaving other individual differences—such as learning strategies, learning styles, and cultural background—largely unexplored. Among these, emotional intelligence, or emotional regulation, deserves particular attention. As a learner's or teacher's power to perceive, express, assess, comprehend, and regulate emotions for the enhancement of emotional and intellectual development, emotional regulation is assumed to have an indirect positive impact on learners' foreign language achievements as well as teachers' teaching achievements mediated by enjoyment. This connects directly to the previous discussion of the mechanisms through which enjoyment operates, generating a new issue of positive emotion intervention that remains unsettled. Scholars have made efforts to intervene in learners' foreign language anxiety from the perspective of cognitive psychology, but how learners and teachers can be guided by themselves or by psychological experts to increase their positive emotions (enjoyment) and alleviate negative emotional experiences in light of positive psychology remains underdocumented and warrants future exploration.

Third, given that the current studies reviewed have mainly focused on face-to-face instructional contexts, online learner enjoyment, a recently raised issue, deserves further and in-depth investigation from distinct perspectives. Regarding learners' online enjoyment, follow-up research is suggested to shed more light on the discrepancy between offline and online enjoyment sources and the influential variables. In particular, how learners' online enjoyment can be strengthened through self-directed or teacher-facilitated strategies, and what approaches can mitigate potential drawbacks such as psychological isolation, decreased concentration, or exposure to incorrect or misleading linguistic knowledge. Furthermore, studies on teachers' enjoyment in online teaching contexts remain scarce, representing an underexplored yet promising avenue for future inquiry.

Fourth, in accordance with the learner enjoyment studies, a set of variables such as gender, age, resilience, grit, engagement, and anxiety that influence FLTE have been investigated. However, the question of how FLTE interacts with various factors to influence foreign language teaching achievement remains largely unexplored. These factors include teachers' individual differences (e.g., international experience, professional title, school level, and personality) and broader social elements (e.g., education policy, salary, and family wellbeing). To address this gap, the perception of multiple interactions of FLTE with these variables should be taken in future research to provide more evidence on teacher enjoyment.

Finally, existing studies have offered valuable insights into overall FLE, yet they have not distinguished between enjoyment derived from learning and teaching different language skills. This dimension deserves further scholarly attention, as levels of enjoyment may differ significantly across listening, speaking, reading, and writing for both learners and teachers. Given the current lack of validated instruments in this area, the development of skill-specific enjoyment scales for both students and teachers would also be a valuable contribution.

## 6 Conclusion

Through the narrative review, this study presents the development of research in relation to enjoyment in FLEd from the perspective of positive psychology in a dozen years past. By and large, two notable research themes about enjoyment in a FLEdal context can be concluded from the previous literature: learners' learning enjoyment and teachers' teaching enjoyment. Based on the valuable findings yielded from the extant studies, the current research status of both two types of enjoyment with focus on their conceptions, measurements, influential factors, dynamic nature and mediating role are tracked and scrutinized in the review. The extensive analysis of these achievements and gaps concerning enjoyment in foreign language learning and teaching environments can not only direct future research to unveil the complexity of human psychological states but also aid foreign language learners and teachers in being aware of the roles of positive emotions in the language scholastic and instructional process to benefit their learning and teaching. Moreover, despite of the fruitful discoveries of enjoyment research in FLEd, future inquiries call for reconceptualization of different types of enjoyment, more conscious theoretical integration and greater methodological diversity to address a string of novel issues advanced.
